# Wheat genotypes with higher yield sensitivity to drought overproduced proline and lost minor biomass under severer water stress

**DOI:** 10.3389/fpls.2022.1035038

**Published:** 2022-11-29

**Authors:** Xinying Zhang, Zhenzhao Wang, Yuzhong Li, Rui Guo, Enke Liu, Xiaoying Liu, Fengxue Gu, Ziguang Yang, Shuying Li, Xiuli Zhong, Xurong Mei

**Affiliations:** ^1^ Key Laboratory of Dryland Agriculture, Ministry of Agriculture, Institute of Environment and Sustainable Development in Agriculture, Chinese Academy of Agricultural Sciences, Beijing, China; ^2^ Hebei Key Laboratory of Crop Stress Biology, College of Agronomy and Biotechnology, Hebei Normal University of Science & Technology, Qinhuangdao, China; ^3^ Crop Stress Resistance Institute, Luoyang Academy of Agriculture and Forestry, Luoyang, China; ^4^ Forestry Institute, Forestry and Grassland Bureau of Aohan Banner, Chifeng, China

**Keywords:** drought, yield loss, proline, metabolomics, water stress, wheat

## Abstract

To clarify the differences in growth and yield responses to drought stress among genotypes contrasting in environmental background, dryland and irrigated genotypes, as well as the underlying biochemical mechanism would provide valuable information for developing superior dryland cultivars. Pot experiments for the whole life cycle in fifteen genotypes and comparative metabolomics analysis for seedlings between two drought tolerant (DT) dryland genotypes and two drought sensitive (DS) irrigated ones were carried out. The DT dryland genotypes suffered heavy biomass loss during severer drought but showed minor yield loss ultimately, while the DS irrigated ones showed minor biomass loss but greater yield loss. Additionally, the superior DT dryland genotypes showed better yield performance under both drought stress and well-watered conditions, indicating their possessing both drought tolerance and high yield potential traits. Suffering severer drought stress, seedling leaves of the DS irrigated genotypes increased some amino acids and organic acids to maintain cell metabolism and accumulate more biomass. Proline in particular was overproduced, which might cause toxicity to cell systems and lead to enormous yield loss ultimately. In contrast, DT dryland genotypes increased the beneficial amino acid and phenolic acids to enhance cell self-protection for alleviating drought damage and efficiently minimized yield loss ultimately.

## 1 Introduction

Wheat (*Triticum aestivum* L.) is one of the major food crops worldwide, but wheat production has been limited by water shortage. In the rain-fed dryland, in particular, drought is a significant stressor with a major impact on plant growth and development, thus cause serious yield losses (Fahad et al., 2017). Increasing the grain yield of wheat in the extensive dryland areas is critical to the world’s food security. To breed superior genotypes targeted towards dryland, named dryland genotypes, that can survive longer and severer drought to gain better and more stable harvest, is the most efficient and sustainable way to reduce drought damage in dryland.

Generally considered, through long-term artificial selection, superior dryland genotypes were conferred strong drought tolerance, and superior genotypes targeted towards irrigated environments, named irrigated genotypes, were conferred high yield potential to obtain good harvest in the high productive environments with ample rainfall or plentiful supplemental irrigation. But a finding obtained by the International Winter Wheat Improvement Program (IWWIP) provides valuable information for winter wheat improvement. IWWIP each year evaluates wheat genotypes across diverse semi-arid and irrigated environments within Central and West Asia and North Africa and upon request beyond. They reported that the highest yielding dryland and irrigated genotypes obtained comparable yield under irrigated management, indicating that certain genotypes developed for dryland also possess yield potential that could efficiently utilize management practices for high productivity (Sharma et al., 2009). This also suggests that the desired traits related to strong drought tolerance and high yield potential could be combined in one genotype.

Based on the above, crop yield in drought environments is not only determined by drought tolerance, but also affected by yield-related traits to a great extent. Moreover, for crop cultivars aimed at economic yield, drought tolerance/susceptibility definitely means high/low yield sensitivity to drought. Thus yield loss after drought stress, rather than yield itself, is more liable for evaluating drought tolerance. That is, after experiencing drought stress, those suffer minor yield loss are drought tolerant (DT) genotypes, and those suffer greater yield loss are drought sensitive (DS) genotypes, as virtually supported by the majority of drought tolerance evaluation systems for cereal crops, in which yield is the key index (Cattivelli et al., 2008). Additionally, wheat plants, grown in no matter dryland or irrigated environments, encounter light to moderate drought from time to time in their life cycle. Distinctly, without supplemental irrigation to protect wheat plants from severer drought damage especially in the key developing phases, wheat plants in dryland environments frequently suffer from longer-lasting and severer drought. Thus, to investigate the differences between wheat genotypes with contrasting water environment backgrounds in terms of growth performances under severer drought, ultimate yield responses, as well as the biochemical mechanisms involved is expected to provide useful information for developing superior dryland cultivars.

When exposed to drought stress, the metabolic homeostasis is destroyed and changed, which involves many dramatic changes in expression of genes and accumulation of proteins that ultimately lead to changes in plant metabolism (Fiehn, 2002; Piasecka et al., 2017) Using metabolomics as a powerful diagnostic tool, great progresses have been made in understanding physiological responses to stress. The most well documented changes in plant metabolism under drought stress are related to the accumulation of certain metabolites, such as amino acids, organic acids, carbohydrates, lipids, terpenoids, etc. Some metabolic changes are common among different stresses, and among different species or genotypes, whereas others are specific. Piasecka et al. (2017) reported that in barley, ferulic and sinapic acid derivatives as well as acylated glycosides of flavones were quite dramatically accumulated; and the polyamine derivatives hordatines along with terpenoid blumenol C derivatives were observed to be drought related. Yang et al. (2018) found in maize that drought stress induced an accumulation of simple sugars and polyunsaturated fatty acids and a decrease in amines, polyamines and dipeptides in a drought-sensitive line (B73); conversely, sphingolipid, sterol, phenylpropanoid, and dipeptide metabolites accumulated in a drought-tolerant line (Lo964). Sanchez et al. (2012) investigated metabolic responses following different levels of drought stress in a Lotus japonicus species, allowing them to identify correlations between the stress level and the magnitude of changes in the metabolite profiles. These researches focused on the effects of drought stress on metabolism, differences in metabolism regulation among genotypes, and among stress intensities. However, how metabolism regulation differed among genotypes with contrasting water environmental background, and how metabolism regulation associates with yield variation have been rarely reported. To analyze the metabolic profiles of the representative DT dryland genotypes and DS irrigated genotypes under severer drought stress is expected to clarify the mechanisms defining the stronger drought tolerance and to find vital indicator metabolite for drought tolerance evaluation.

The North China Plain is the most important winter wheat production region in China, supplying about 75% of China’s wheat (National Bureau of Statistics of China, 2021). Ten irrigated and five dryland genotypes from this region were selected to investigate the growth performances under severer drought stress and the ultimate grain yield responses. Thereafter, two DT dryland genotypes and two DS irrigated genotypes were selected for analyzing biochemical mechanism underlying the differential drought responses by high-throughput targeted metabolomics. The major objectives of this study was to 1) link the growth performance during severer drought stress at vegetative stage to ultimate yield response; 2) reveal how the DT and DS wheat types targeted towards contrasting water environments differed in metabolism regulation responding to severer drought; 3) analyze the vital metabolites involved in drought tolerance and crops production, especially at an early stage and in the method independent of natural droughts, which would help crop breeders in specific selection programmes.

## 2 Materials and methods

### 2.1 Experiments for investigating growth and yield performances responding to drought stress

#### 2.1.1 Plant materials

Fifteen winter wheat genotypes from the North China Plain, including five dryland and ten irrigated ones, were selected from a wheat germplasm nursery affiliated to Luoyang Academy of Agriculture and Forestry, located in the central region of the North China Plain. Dryland genotypes were two released cultivars Jinmai 47 and Chang 6878, and three unreleased lines 908216, 908032, and 908206. Irrigated genotypes were Shijiazhuang 8, Lankaoaizao 8, Zhengmai 9023, Yumai 18, Zhoumai 18, Zhoumai 14, Jiaomai 266, Yanzhan 4110, 12 Song, and Jing 411. For selecting wheat materials, stomotal conductance (Gs) was measured in a large number of genotypes during the period time of 9 - 10 o’clock at jointing stage under rain-fed and irrigated conditions. According to the data collected, the 15 genotypes in a wide spectrum of Gs from very low to rather high value in the two water conditions were selected as materials.

#### 2.1.2 Growth conditions

**Figure 1:**
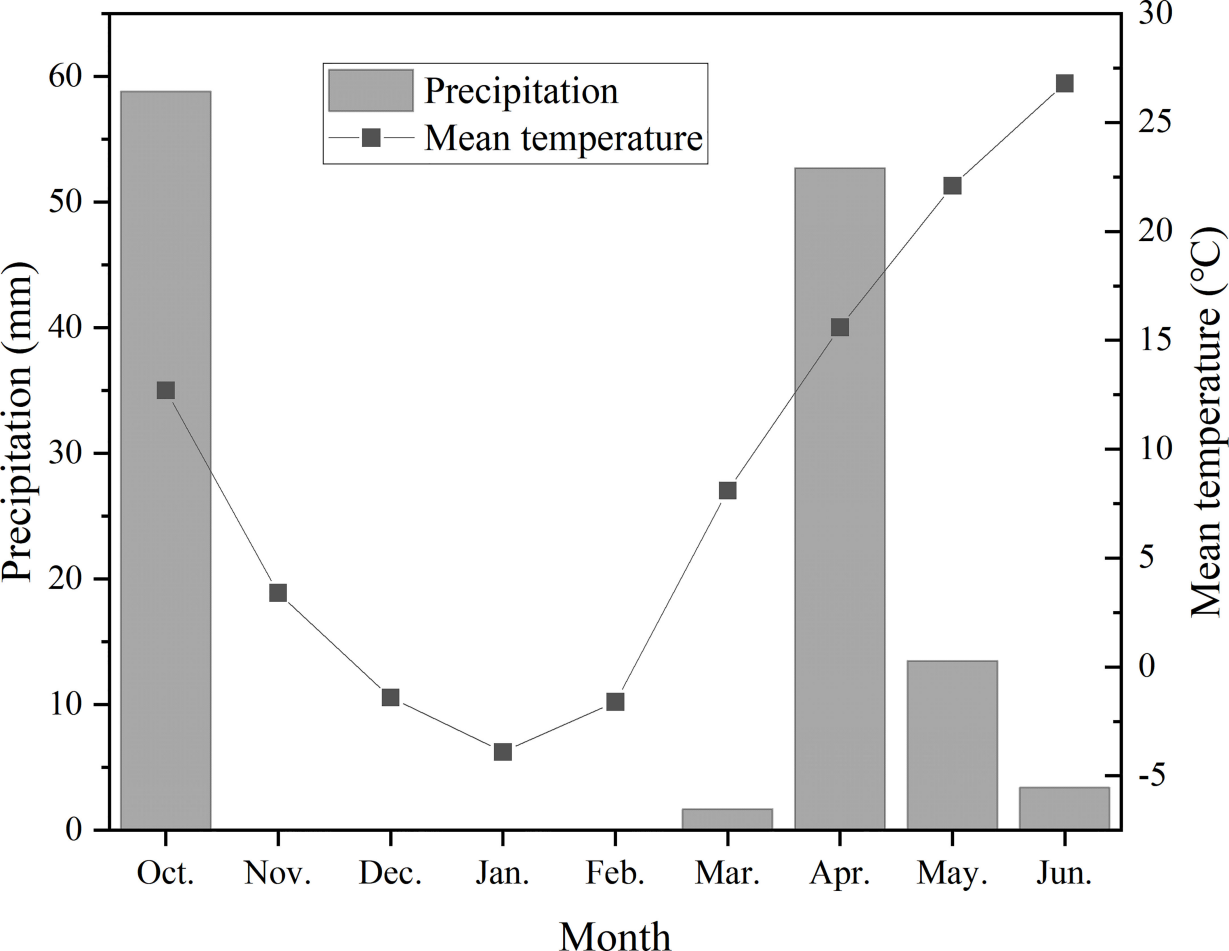
Monthly precipitation and monthly mean air temperature during winter wheat growing seasons. *Precipitation in October was sum from the sowing day to October 31. Precipitation in June was sum from June 1 to the harvesting day.

The experiments were conducted at Shunyi Scientific Experimental Station, affiliated to Institute of Environment and Sustainable Development in Agriculture, Chinese Academy of Agricultural Sciences (CAAS) in 2017-2018 growing seasons of winter wheat. Pot cultivation was adopted in this experiment. Polyvinyl chloride (PVC) pots were 20 cm in depth and 35 cm in diameter, with a drainage hole on the bottom. The pots were filled with 16 kg plow layer soil, which was sieved through a 5 mm sieve and then fully mixed. The soil nutrients were determined as 0.109 g/kg of total nitrogen, 14.4 g/kg of organic matter, 24.5 mg/kg of available phosphorus, 106 mg/kg of available potassium, and the soil pH was 7.7. The monthly mean air temperature and monthly precipitation for the experimental growing season are shown in **Figure 1**.

The sowing date was October 5, 2017. Twenty wheat seeds were sown in each pot. Thirteen uniform seedlings were kept for overwintering, with the slender ones being removed 30 d after emergence. After recovering in the spring, 10 uniform seedlings were eventually selected and kept as materials, with the unqualified ones being eliminated. For each genotype, 6 pots were planted for two water treatments and three replications.

#### 2.1.3 Water treatments

Two water treatments were arranged: well-watered (WW) and water stress (WS) conditions, with soil water content being 75% and 50% of field water holding capacity (FC), respectively. To prevent different soil water content in the pots caused by differing transpiration rate among genotypes and ensure the plants of each genotype were treated with uniform drought intensity, water withholding was conducted by the weighing method. The plants were watered daily to restore the initial soil water content, between 1 and 2 h after sunset. Water withholding was kept for 30 d from April 6 through May 6. During water treatment periods, a rain-shelter was utilized to prevent rain on rainy days and was kept rolled up on sunny days to allow the crops to grow in the open-air conditions.

#### 2.1.4 Measurement of biomass accumulation and grain yield

The wheat plants under WW and WS conditions were randomly sampled on April 10 and April 30, 2018. The samples were placed in the blast oven at 105 °C for 30 min, then dried at 75 °C for 48 h until they reached a constant weight, then the dry matter was weighed. Biomass accumulation was calculated as the difference in dry weight between samples taken on the two dates. After matured, the plants were harvested by artificial sickle cutting method, then put in net bags and air-dried. When they reached a constant weight, the plants were artificially threshed and the grain yielded was weighed.

#### 2.1.5 Measurement of net photosynthetic rate (Pn)

The youngest fully expanded leaves of the main tiller were measured at 9:30-11:00 AM on April 29, 2018, utilizing a Licor-6400 portable infra-red gas analyzer (LI-COR Inc., Lincoln, NE, USA). The leaf chamber conditions were set at the temperature of 25 °C, PAR of 1,000 mmol·m−2·s−1. For each replication, the leaves of three plants were measured and averaged.

### 2.2 Experiments for metabolomics analysis of seedlings under severer drought stress

#### 2.2.1 Plant materials and growth conditions

Based on their contrasting growth and yield performances to drought, two DT dryland genotypes Jinmai 47 and 908216, and two DS irrigated genotypes Lankaoaizao 8 and Zhengmai 9023 were selected for further metabolomics analysis. Before germination, seeds were rinsed with distilled water after being soaked in 10% sodium hypochlorite solution for 10 min, then distributed in a 90 mm-diameter sterile Petri dish with two layers of filter paper soaked with distilled water at the bottom. The petri dishes were placed in a climate incubator, with 25 °C, 50% relative humidity, and no light for 4 days. About 5 ml of distilled water was added to the surface of seeds every day. Then, seeds with the same buds were transferred to 17 cm diameter plastic pots (12 seeds per pot). The pots were filled with 850 g soil and placed in a growth chamber (16h/8h photoperiod at day/night, temperature 25°C/15°C, photosynthetically active radiation 700 μmol·m^−2^·s^−1^ and relative humidity 45-55%), which can provide a more stable environmental condition than the field.

#### 2.2.2 Drought stress treatment

Two-week-old wheat seedlings with 2 fully expanded functional leaves were exposed to progressive drought stress. WW group was watered daily to make the soil water content reach 75% of the field water holding capacity by the weighing method. Throughout the stress time course, the Pn of seedlings had been monitored. The measurement method for Pn and other photosynthetic parameters was similar to the above, with only PAR being modified to 800 μmol·m^−2^·s^−1^. On the tenth day of treatment, when Pn of the wheat genotypes leaves was approaching zero, the third fully expanded leaves were sampled. On the same days, leaves from WW plants were sampled. All samples were collected in three biological replicates. The harvested leaf samples were immediately frozen in liquid nitrogen for 30 min and stored at −80°C until metabolite extraction and instrumental analysis.

#### 2.2.3 Metabolite extraction and LC-MS analysis

##### 2.2.3.1 Sample extraction process

800mg samples were vacuum-freeze-dried in a lyophilizer (scientz-100f). The freeze-dried sample was crushed using a mixer mill (MM 400, Retsch) for 1.5 min at 30 Hz. 100 mg powder was weighed and extracted overnight at 4°C with 1.2 ml 70% aqueous methanol, which was vortexed six times to improve the extraction rate during this period. Following centrifugation at 12000 rpm for 10 min, the extracts were filtrated (0.22 μm pore size) and stored in the sample bottle.

##### 2.2.3.2 Chromatography and mass spectrometry acquisition conditions

Data acquisition instrument system mainly includes ultra-performance liquid chromatography system (UPLC) (SHIMADZU Nexera X2, https://www.shimadzu.com.cn/) and tandem mass spectrometry (MS/MS) (Applied Biosystems 4500 QTRAP, http://www.appliedbiosystems.com.cn/). The liquid phase condition was as follows, UPLC column, Agilent SB-C18 (1.8 µm, 2.1 mm*100 mm); The mobile phase consisted of solvent A, pure water with 0.1% formic acid, and solvent B, acetonitrile with 0.1% formic acid. Sample measurements were performed with a gradient program that employed the starting conditions of 95% A, 5% B. Within 9min, a linear gradient to 5% A, 95% B was programmed, and a composition of 5% A, 95% B was kept for 1min. Subsequently, a composition of 95% A, 5.0% B was adjusted within 1.10 min and kept for 2.9 min. The flow rate was 0.35 ml/min; the column oven was set to 40°C; the injection volume was 4μl. Mass spectrometry conditions mainly include: ESI (electrospray ionization, ESI) source operation source temperature 550°C; ion spray voltage 5500 V (positive ion mode)/-4500 V (negative ion mode); curtain gas (CUR) 25.0 psi; the collision-activated dissociation (CAD) was high. In the triple quadrupole (QQQ), each ion pair is scanned according to the optimized declustering potential (DP) and collision energy (CE) (Chen et al., 2013).

##### 2.2.3.3 Qualitative and quantitative analysis of metabolites

Based on MWDB (Metware Database), the material was qualitative according to secondary spectrum information. Isotope signals, repeated signals containing K^+^ ions, Na^+^ ions and NH_4_^+^ ions, as well as repeated signals of fragments of other substances with larger molecular weight were removed during analysis. The metabolite quantification was accomplished by using the multiple reaction monitoring (MRM) mode of triple quadrupole mass spectrometry. In the MRM mode, the precursor ions (parent ions) of the target substance were firstly screened by the quadrupole, and excludes the ions corresponding to other molecular weight substances to eliminate interference initially; the precursor ions are induced by the collision chamber to ionization and then break to form many fragmented ions, and the fragmented ions are then filtered through the triple quadrupole to select the required characteristic fragment ions, excluding non-target ion interference, making the quantification more accurate and the reproducibility better. After obtaining the metabolite mass spectrometry analysis data of different samples, the peak area integration of all substance mass spectrometry peaks is performed, and the mass spectrometry peaks of the same metabolite in different samples are integrally corrected (Fraga et al., 2010).

### 2.3 Data analysis

The collected data of biomass accumulation and grain yield were statistically analyzed with the least significant difference method (LSD) by SAS software (SAS 9.4, Cary, NC, USA), and significance was considered at P < 0.05. The metabolome data was analyzed with orthogonal partial least squares-discriminant analysis (OPLS-DA) model, orthogonal variables that are not related to categorical variables in metabolites are filtered out, and non-orthogonal variables and orthogonal variables are analyzed separately, in order to better classify the two types of genotypes under WW and WS and obtain more reliable differential metabolites. This study mainly uses the R (3.3.2) package ropls to calculate the OPLS-DA model. Differential metabolites were screened by the method of combining multiple of difference, P-value of T-test and VIP value of OPLS-DA model, and the screening criteria were FC>1, P value<0.05 and VIP>1.

## 3 Results

### 3.1 DS genotypes with high yield sensitivity to drought conversely showed better growth performances under severer drought stress

Under WW condition, the 15 tested genotypes showed different yield performances. Shijiazhuang 8, a famous irrigated genotype, gained 8.64 g/plant of yield, ranking the top, and Zhoumai 18, also irrigated genotype, obtained 7.08 g/plant of yield, ranking the bottom. All the five dryland genotypes showed superior yield performance of more than 8.40 g/plant (**Table 1**).

**Table 1:** 

Groups	Genotypes	Biomass (g/plant)			Grain yield (g/plant)			Photosynthetic rate(µmol·m⁻²·s⁻¹)	
		WW	WS	BiomassReduction (%)	WW	WS	YieldLoss (%)	WW	WS
**I** (Dryland genotypes)	908206	3.51cd	2.44de	30.53bc	8.45abc	7.78ab	7.95a	22.11efg	7.93fg
	908032	3.56c	2.34ef	34.31ab	8.42bc	7.74ab	8.05a	23.21bcde	8.67ef
	908216	3.63bc	2.29f	36.9a	8.47abc	7.76ab	8.37a	22.94bcde	8.36ef
	Jinmai 47	3.68bc	2.32ef	37.01a	8.54ab	7.82a	8.42a	23.00bcde	8.06fg
	Chang 6878	3.68bc	2.42de	34.11ab	8.51abc	7.59b	10.84ab	21.22g	7.29g
**II** (Irrigated genotypes)	Jiaomai 266	3.3de	2.43de	26.45cde	7.67e	6.68d	12.9bc	21.45fg	11.72bc
	Jing 411	3.54c	2.67b	24.52efg	8.31c	7.15c	13.97bc	23.5abcd	12.24abc
	Zhoumai 18	3.21ef	2.13g	33.48ab	7.08h	6.03fg	14.69c	23.81abc	9.24de
**III** (Irrigated genotypes)	Yumai 14	2.97g	2.52cd	15.12h	7.42fg	6.07f	18.25d	23.15bcde	11.43bc
	Yanzhan 4110	3.02fg	2.25fg	25.5def	7.25gh	5.84g	19.41d	22.62cdef	11.57bc
	12 song	3.84ab	2.97a	22.68efg	7.88d	6.23ef	20.89de	24.56a	12.89a
**IV** (Irrigated genotypes)	Yumai 18	3.27e	2.61bc	20.18g	8.41bc	6.43e	23.53ef	23.1bcde	11.34c
	Lankaoaizao 8	4.03a	3.05a	24.23efg	7.54ef	5.62h	25.81f	24.18ab	12.37ab
	Zheng 9023	3.72bc	2.95a	20.77fg	7.58ef	5.16i	31.64g	24.72a	13.08a
	Shijiazhuang 8	3.78b	2.64bc	30.11bcd	8.64a	5.24i	39.35h	22.39defg	9.69d

Values with different letters in the column indicated significant difference at P <0.05 level. Dryland genotypes Jinmai 47 and 908216 (in orange color shading) strongly contrasted with irrigated genotypes Lankaoaizao 8 and Zheng 9023 (in green color shading) in biomass reduction and yield loss responding to severer drought stress.

With drought tolerance being evaluated in the light of yield loss under WS condition, the 15 genotypes were classified into four groups. Group I genotypes, in which the 5 dryland genotypes were included, showed yield loss less than 10%, thereby were the most DT group. And Group IV genotypes, showing more than 23% of yield loss, were the most DS group (**Table 1**).

Interestingly, biomass performances during drought stress were exactly the opposite of yield responses for the DT and DS genotypes. Twenty-day drought stress caused biomass loss in DT genotypes as high as more than 30%, with the loss reaching 37.01% and 36.9% in Jinmai 47 and 908216 separately. While biomass loss was less than or close to 25% for most of the DS genotypes, being only 15.12% in Yumai 14. Pn of genotypes leaves was determined at the end of drought treatment. Similarly, Pn was lower in DT than in DS genotypes generally (P<0.05), consistent with biomass variation trend, explaining well the different biomass performances between the two types of wheat (**Table 1**).

### 3.2 Seedlings of the DS irrigated genotypes showed higher Pn than DT dryland genotypes under severer drought

**Figure 2:**
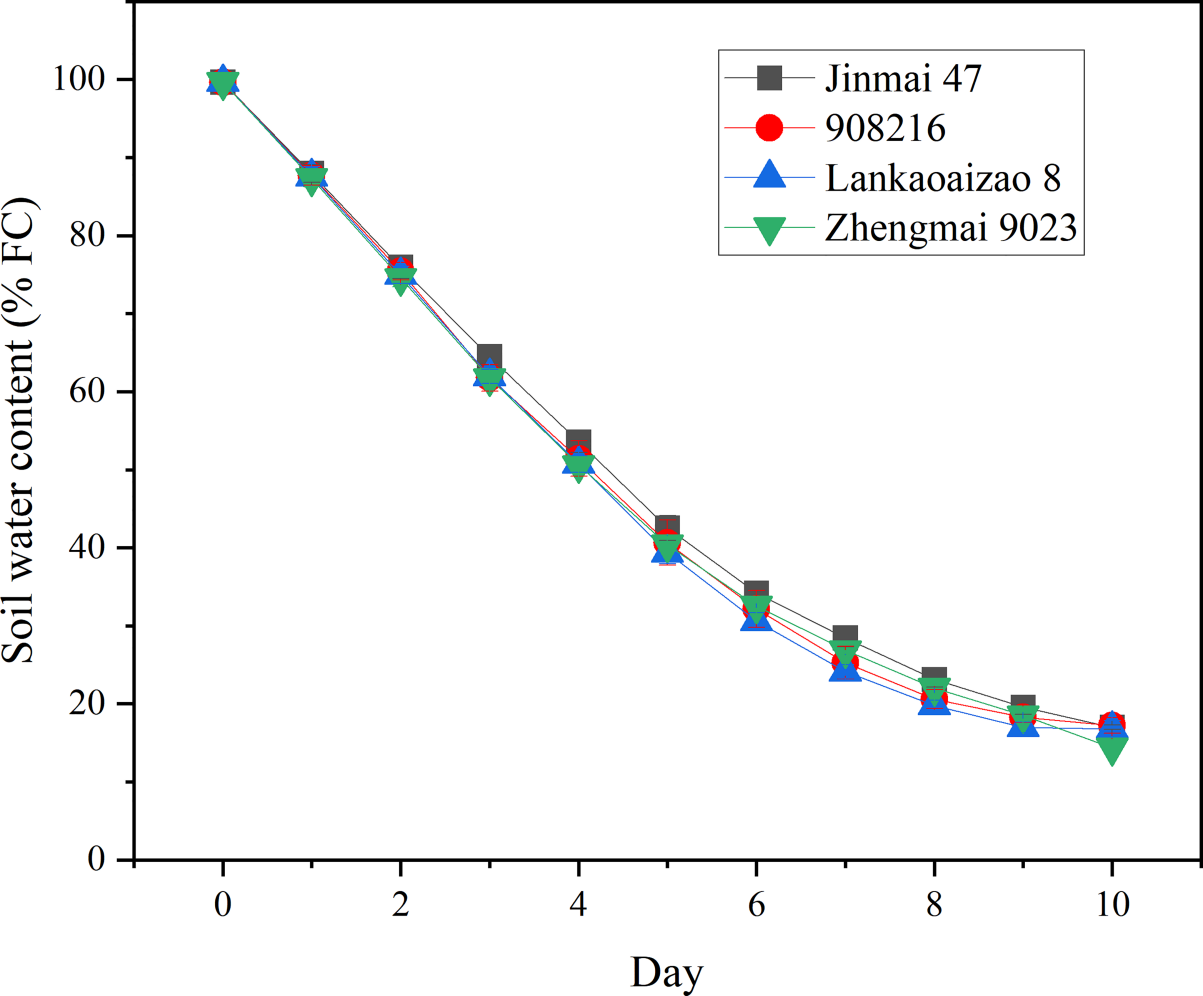
The dynamic changes in soil water content of the four genotypes exposed to progressive drought stress. The soil water content refers to the percentage of field water holding capacity (FC). Values represent means ± standard errors (n=3).

**Figure 3:**
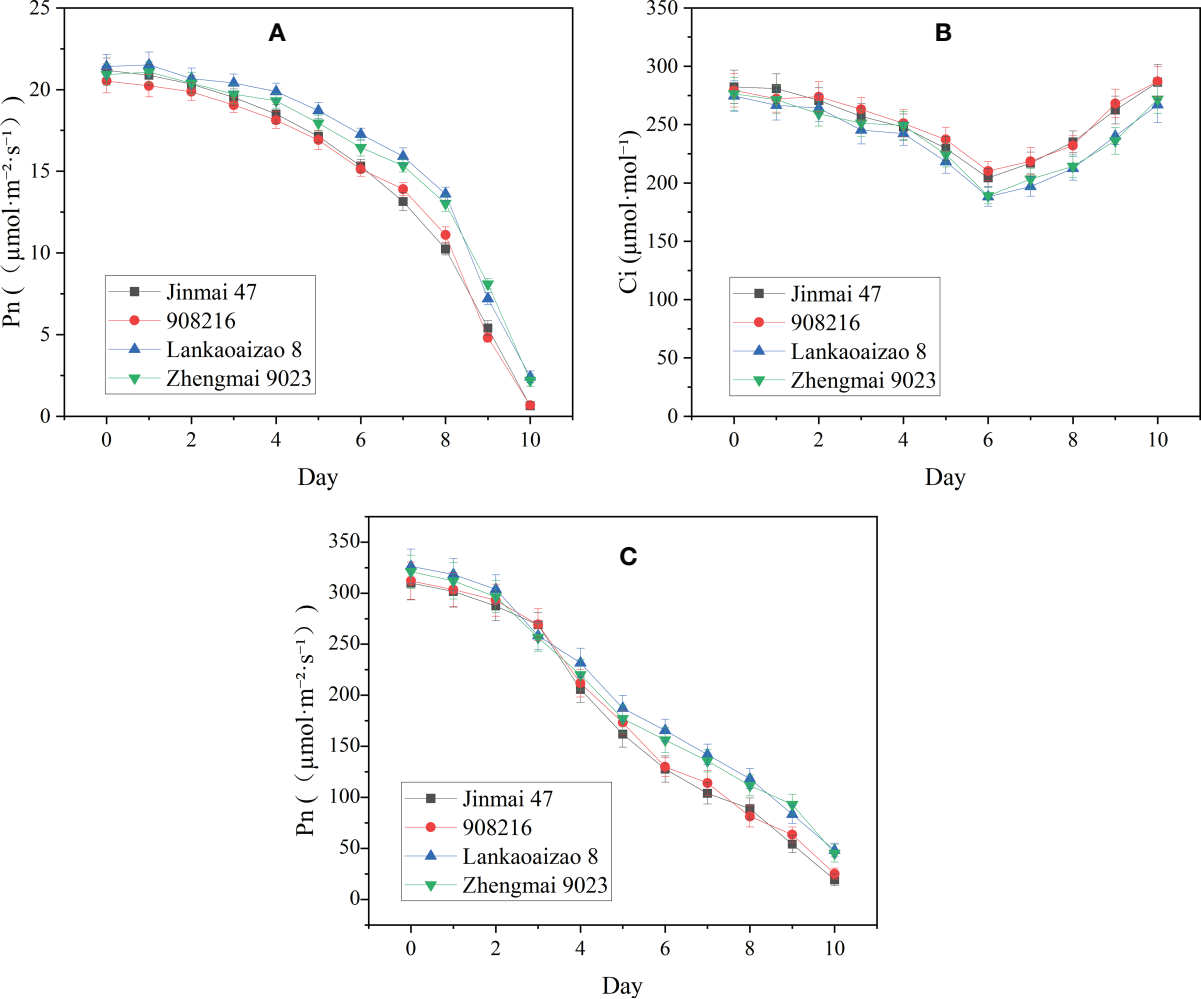
The dynamic changes in photosynthetic parameters of the four genotypes exposed to progressive drought stress. **(A)** Pn, net photosynthetic rate; **(B)** Ci, concentration of intercellular CO_2_; **(C)** Gs, stomotal conductance. Values represent means ± standard errors (n=3).

Based on their contrasting growth and yield responses to drought, two DT dryland genotypes (Jinmai 47 and 908216) and two DS irrigated genotypes (Lankaoaizao 8 and Zhengmai 9023) were selected for the further analysis on their metabolic responses to drought. Throughout the stress time course, soil water content and Pn of seedlings had been monitored (**Figures 2** and **3**). During 10-day progressive drought stress, Pn and Gs in the four genotypes gradually came down up to lower than 2 µmol·m⁻²·s⁻¹ and lower than 20 mol·m⁻²·s⁻¹ respectively with stress time prolonging. Within 5 d of treatment, there were no significant differences in Pn and Gs between the DT and DS genotypes (**Figure 3**). This indicated that the two types of wheat did not differ in photosynthetic parameters under light to moderate drought stress. Six days after the onset of stress, when soil water content decreased to 32% of FC or so (**Figure 2**), the concentration of intercellular CO_2_ (Ci) reached the bottom, and was turning to increase (**Figure 3**). Ci rise indicated that severer drought has caused damage to photosynthetic organs and the related enzymes, which turned to be the primary factor limiting photosynthetic rate. Right from Ci turning, the DT and DS genotypes significantly differed in Pn, Gs, and Ci. Pn and Gs were lower, and Ci was higher in the two DT genotypes Jinmai 47 and 908216 than in the two DS genotypes Lankaoaizao 8 and Zhengmai 9023 (P<0.05).

### 3.3 The DT dryland genotypes and the DS irrigated genotypes showed contrasting metabolic regulation in response to severer drought stress

#### 3.3.1 Metabolomics analysis of wheat seedlings for the DT and DS genotypes under severer drought stress

**Figure 4:**
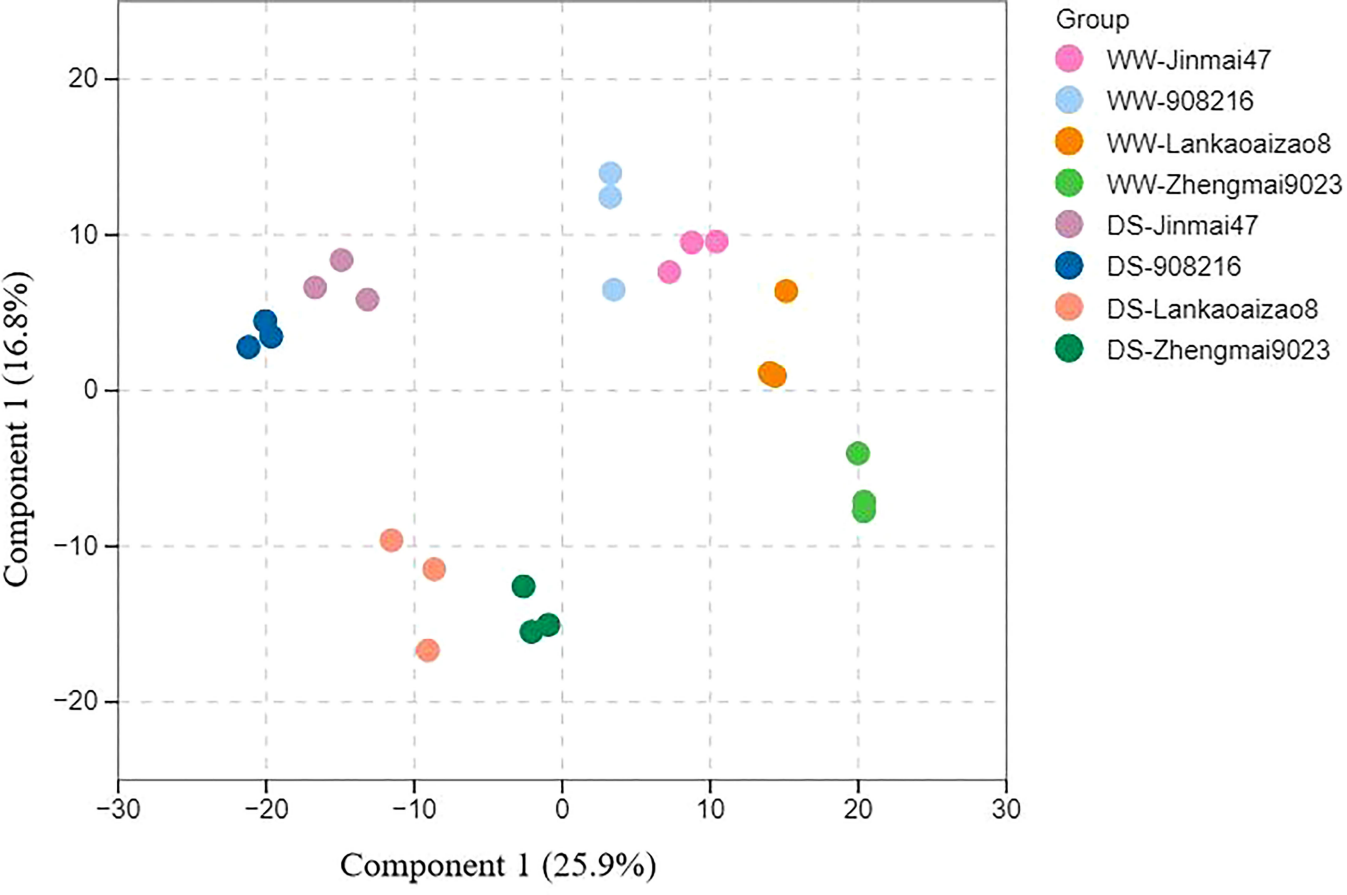
Metabolome analysis of the four wheat genotypes leaves under well-watered condition (WS) and water stress condition (WW). The data is analyzed by orthogonal partial least squares-discriminant analysis. Component 1: the first principal component, Component 2: the second principal component. Three biological replicates (n=3) were set up for each treatment.

Seedling leaves of DT genotypes Jinmai 47 and 908216 and DS genotypes Lankaoaizao 8 and Zhengmai 9023 under severer drought stress were subjected to metabolomics analysis. The metabolic profiles for the four genotypes were obtained based on UPLC-MS/MS platform. A total of 731 metabolites were identified, and their corresponding concentrations were determined. These detected metabolites are distributed into different classes, including amino acids and their derivatives, organic acids, phenolic acids, lipids, sugars and sugar alcohols, lignans and coumarins, alkaloids, terpenoids and so on (**Table S1**). The four wheat genotypes under different treatments were not well separated through principal component analysis of their metabolites. The partial least squares-discriminant analysis (OPLS-DA) was adopted to determine the difference among the four wheat genotypes under WW and WS conditions. The first two components of OPLS-DA explained 42.7% of the variation and highlighted the distinct clustering between DT and DS wheat genotypes and two soil water conditions, which suggested that differential metabolite accumulation patterns accounted for the variation (**Figure 4**).

**Figure 5:**
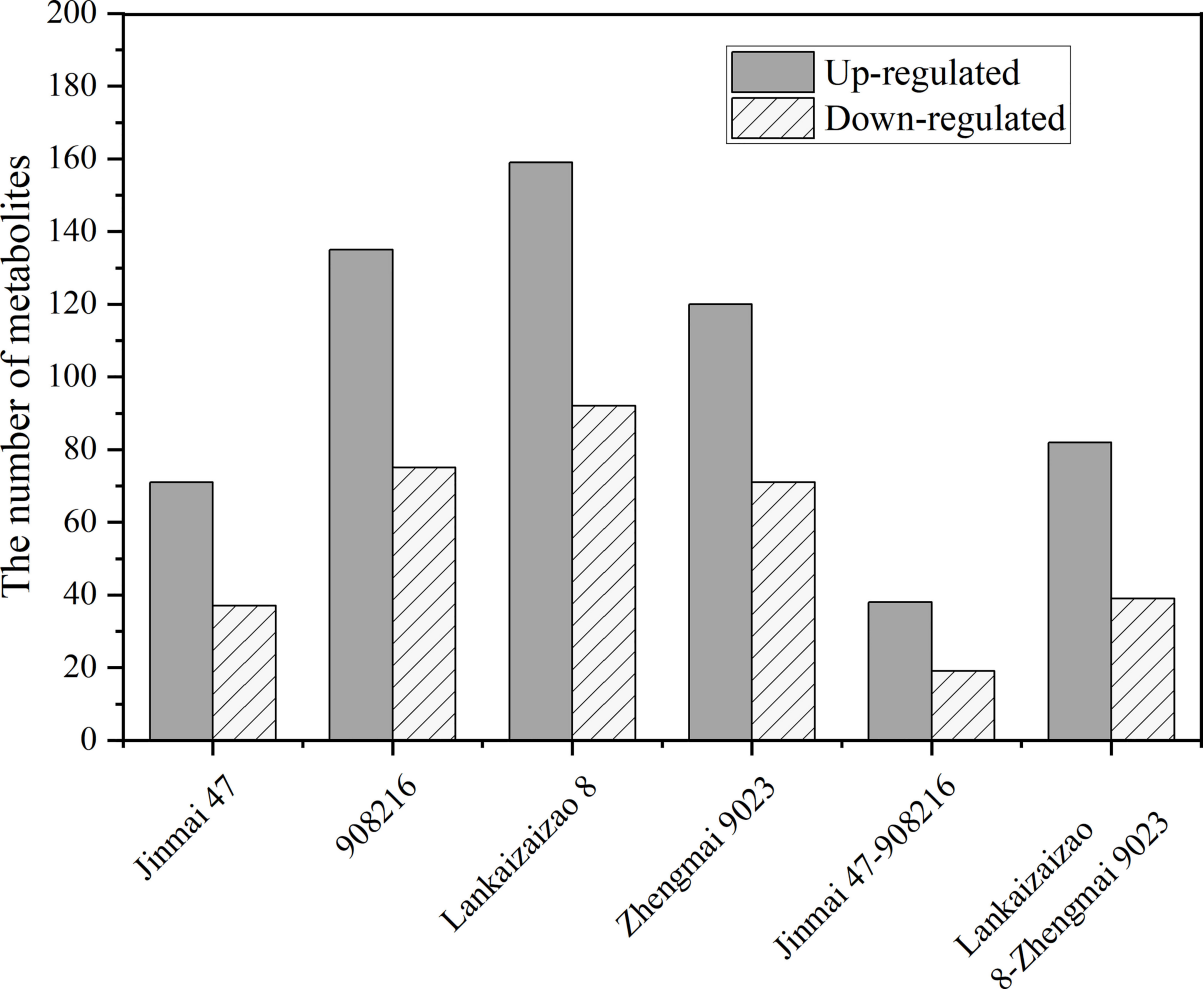
The number of significantly up-regulated or down-regulated metabolites under drought stress in the four genotypes Jinmai 47, 908216, Lankaoaizao 8, and Zhengmai 9023 respectively, as well as that in the two drought tolerant dryland genotypes Jinamai 47 and 908216 in common, and that in the two drought sensitive irrigated genotypes Lankaoaizao 8 and Zhengmai 9023 in common.

Exposed to drought treatment, the both DT genotypes Jinmai 47 and 908216 showed 38 up-regulated and 19 down-regulated metabolites in common, and the two DS genotypes Lankaoaizao 8 and Zhengmai 9023 had 82 up-regulated and 39 down-regulated metabolites in common (**Figure 5**). These metabolites were broadly classified into amino acids, organic acids, phenolic acids, lipids, and others, and then subjected to fold-change analysis to describe direction and intensity of regulation, and to figure out the significantly different metabolites between DT and DS genotypes.

#### 3.3.2 Differences in metabolic regulation responding to severer drought stress between the DT and the DS genotypes seedlings

Some amino acids and organic acids were significantly increased in the two DS irrigated genotypes under severer drought stress (**Table 2**). The most pronounced amino acid up-regulated was proline. Although all the 4 genotypes showed remarkable up-regulation of proline, the DT and DS genotypes strongly contrasted in the regulation magnitude. It was increased by 25.43-fold and 14.13-fold in the DS genotypes Lankaoaizao 8 and Zhengmai 9023 respectively, while increased by only 8.96-fold and 9.00-fold in the DT genotypes Jinmai 47 and 908216 respectively. While in term of relative content, it might not affirm that the two types of wheat, DS and DT genotypes, differed from one another, since no significant difference existed between 90816 and Zhengmai 9023 (**Table S2**). Following proline, L-threo-3-methylaspartate and glutamic acid were significantly up-regulated by up to 3.97-fold and 2.13-fold respectively in Lankaoaizao 8, and 2.96-fold and 2.42-fold respectively in Zhengmai 9023, but did not change significantly in the two DT genotypes. Phenylalanine was the only amino acid up-regulated in the two DT genotypes, but was detected unchanged in the two DS genotypes. Besides, two kinds of organic acids trans-citridic acid and malic acid were up-regulated in the two DS genotypes. Malate is involved in stomatal regulation as known. These up-regulated amino acids and organic acids enhanced cellular osmotic adjustment to maintain photosynthesis and other cellular metabolism, thus might explain the larger biomass accumulation of the DS genotypes under severer drought. On the other hand, overproduced proline and up-regulation of the other metabolites might be damage indicator, since moderate response of proline and no significant change in the other metabolites were observed in the two DT dryland genotypes, which lost minor yield ultimately.

**Table 2:** Fold changes of the important metabolites differing between the drought tolerant dryland genotypes Jinmai 47 and 908216 and the drought sensitive irrigated genotypes Landkaoaizao and Zhengmai 9023 in response to drought stress.

Metabolites Class	Name	Jinmai 47	908216	Lankaizaizao 8	Zhengmai 9023
**Amino acids**	Proline	8.96 ↑	9.00 ↑	25.43 ↑	14.13 ↑
	Threo-3-Methylaspartate	ns	ns	3.97 ↑	2.96 ↑
	Glutamic acid	ns	ns	2.13 ↑	2.42 ↑
	Threonine	ns	ns	0.68 ↓	0.55 ↓
	Valyl-L-Phenylalanine	ns	ns	0.74 ↓	0.78 ↓
	Aspartic Acid	ns	ns	0.56 ↓	0.63 ↓
	Phenylalanine	1.49 ↑	1.76 ↑	ns	ns
	N-Acetyl-L-tyrosine	0.68 ↓	0.67 ↓	ns	ns
**Organic acids**	Trans-Citridic acid	ns	ns	1.35 ↑	1.71 ↑
	Malic acid	ns	ns	1.29 ↑	1.35 ↑
	3-Hydroxyanthranilic acid	ns	ns	0.26 ↓	0.63 ↓
	2,2-Dimethylsuccinic acid	ns	ns	0.25 ↓	0.39 ↓
**Phenolic acids**	Caffeic acid	4.45 ↑	5.54 ↑	2.09 ↑	4.78 ↑
	Ferulic acid	2.32 ↑	1.69 ↑	ns	ns
	Salicylic acid	1.59 ↑	1.87 ↑	ns	ns
	Syringaldehyde	1.59 ↑	1.35 ↑	ns	ns
	4-MethoxycinnaMaldehyde	1.18 ↑	1.23 ↑	ns	ns
	Benzamide	1.65 ↑	1.44 ↑	ns	ns
	3,4-Dihydroxybenzeneacetic acid	ns	ns	1.67 ↑	1.63 ↑
	Vanillin	ns	ns	1.42 ↑	1.40 ↑
	Coniferyl alcohol	ns	ns	2.55 ↑	2.44 ↑
	Methyleugenol	0.43 ↓	0.47 ↓	ns	ns
**Lipids**	9-Hydroperoxy-10E,12,15Z-octadecatrienoic acid	ns	ns	0.60 ↓	0.71 ↓
	Elaidic acid	ns	ns	0.65 ↓	0.77 ↓
	Eicosadienoic acid	ns	ns	0.50 ↓	0.57 ↓
	13S-Hydroperoxy-6Z,9Z,11E-octadecatrienoic acid	ns	ns	0.60 ↓	0.70 ↓
	LysoPC 20:2	ns	ns	0.81 ↓	0.62 ↓
	LysoPE 20:2	ns	ns	0.40 ↓	0.36 ↓
**Others**	4-Methyl-5-thiazoleethanol	ns	ns	2.11 ↑	2.16 ↑
	Pinoresinol-4,4'-O-di-O-glucoside	ns	ns	1.72 ↑	2.51 ↑
	Jaceosidin	ns	ns	1.47 ↑	1.44 ↑
	rhamnoside	ns	ns	1.44 ↑	1.55 ↑
	Salcolin A	ns	ns	1.44 ↑	1.48 ↑
	Choline	ns	ns	1.30 ↑	1.19 ↑
	Adenosine 5'-monophosphate	ns	ns	0.71 ↓	0.49 ↓
	Phenethylamine	1.65 ↑	1.43 ↑	ns	ns

“ns” means that the change of each metabolite is not significant under drought stress in every genotypes, ↑ means significant up-regulation, ↓ means significant down-regulation.

DT dryland genotypes enhanced biosynthesis of some phenolic acids in response to severer drought stress. Phenolic acid is a kind of organic acid containing phenolic rings. Although the two DS genotypes up-regulated three phenolic acids vanillin, coniferyl alcohol, and 3,4-Dihydroxybenzeneacetic acid under severer drought stress, in view of the number and the response magnitude of the regulated metabolites, obviously the two DT genotypes enhanced biosynthesis of phenolic acids predominately. There were six phenolic acids significantly up-regulated in the two DT genotypes (**Table 2**). Of them, five phenolic acids ferulic acid, benzamide, salicylic acid, syringaldehyde, and 4-methoxycinna maldehyde remained unchanged in the two DS genotypes. Caffeic acid was up-regulated in both DT and DS genotypes, and did not show obvious difference in term of regulation fold, but its relative content was significantly higher in the two DT genotypes than in the two DS genotypes (**Table S2**). Phenolic acids could facilitate lignin synthesis to alleviate the damage of drought stress. Thereby this might indicate that the DT genotypes enhanced cell self-protection under severer drought by increasing biosynthesis of phenolic acids.

The DS genotypes significantly down-regulated some lipids. The following six lipids elaidic acid, 9-Hydroperoxy-10E,12,15Z-octadecatrienoic acid, eicosadienoic acid, lysophosphatidyl choline, lysoPC 20:2, and lysoPE 20:2 were down-regulated in the two DS genotypes under drought stress (**Table 2**). These fatty acids and lysolipids are all components of membrane lipids. Down-regulation of these lipids indicated more serious membrane damage caused by severer drought. However, all the six lipids showed no significant variance in the two DT genotypes, indicating their better cellular membrane stability under severer drought stress.

In addition to the main classes of metabolites described above, there were eight other metabolites significantly differing between the DT and DS genotypes (**Table 2**). Of them, the six metabolites 4-Methyl-5-thiazoleethanol, pinoresinol-4,4’-O-di-O-glucoside, jaceosidin, rhamnoside, salcolin A, and choline were up-regulated, and one metabolite adenosine 5’-monophosphate was down-regulated in DS genotypes, while all the seven metabolites remained unchanged in the two DT genotypes. There was only one metabolite phenethylamine that was up-regulated in the two DT genotypes, but did not significantly change in the two DS genotypes.

## 4 Discussion

### 4.1 DS genotypes showed minor biomass loss, higher Pn during severer drought, but suffered greater yield loss ultimately

As mentioned above, for crop cultivars targeted towards economic yield, drought tolerance evaluation necessarily take yield response to drought into account. In the current study, genotypes Zhoumai 18, 12 song, and Yumai 14 gained similar yield under WS condition, but the yield losses were 14.69%, 20.89%, and 18.25% respectively compared with that under WW condition. Crop yield in drought environment depends on both drought tolerance and yield-related traits of a cultivar. However, crop yield loss after suffering drought stress is mostly determined by drought tolerance. Thus in view of yield loss, Zhoumai 18 was stronger in drought tolerance than 12 song and Yumai 14. Of all the tested genotypes, the five dryland genotypes showed rather less yield loss after severer drought treatment, demonstrating their stronger drought tolerance.

Shijiazhuang 8, the typical irrigated genotype, gained the highest yield under WW condition, but lost up to 39.35% of yield suffering drought stress, demonstrating the superior yield potential but rather higher yield sensitivity to drought. While irrigated genotype Jing 411 obtained higher yield under WW condition, also lost less yield after experiencing severer drought stress, showing both higher yield potential and stronger drought tolerance. On the other hand, the five dryland genotypes not only showed rather less yield loss after suffering severer drought stress, but also gained as high yield as the excellent irrigated genotypes under high productive environment, indicating the possession of both high yield potential and strong drought tolerance. Consistently, Sharma et al. (2009) reported 6 out of 20 highest yielding dryland genotypes showed superior performance both under low and high productive environments, demonstrating specific adaptability and yield plasticity. The performances of these genotypes suggested the independency and co-evolution of yield-related and drought tolerance-related traits. It thus supports that superior dryland genotypes could be developed by improving irrigated genotypes to enhance environmental adaptability and yield stability through breeding and selection.

Unexpectedly, most DT genotypes conversely showed more biomass loss and lower Pn than DS genotypes suffering severer drought stress (**Table 1**). Thus a better performance of biomass accumulation during drought stress could not predict a better performance of yield for the wheat genotypes, nor could a higher Pn in the stress period. That is, neither biomass accumulation nor Pn could be used as a liable surrogate for evaluating yield performance responding to drought. More than that, physiological indices raised same questions. Proline level was considered as an important indicator for evaluating various stress tolerance in some cases (Hayat et al., 2012). However, here it was in proline response magnitude that the DT and DS genotypes significantly differed, nor in proline content. The relative content of proline did not differ between DT 908216 and DS Zhengmai 9023 under severer drought (**Table S2**). What’s more, it was the two DS genotypes Lankaoaizao 8 and Zhengmai 9023 that more dramatically increased proline. Thus some evaluation indices could only be used for limited genotypes adapting to similar ecological environments. For genotypes with contrasting water environmental background, evaluation system with yield being the core index remains to be the most robust up to date.

### 4.2 Seedlings of DS genotypes with high yield sensitivity to drought overproduced proline under severer drought stress

Proline is one of the most documented compatible compounds up to now. It plays highly beneficial roles in plants exposed to various stress conditions, i.e. as an excellent osmolyte maintaining cell hydration and turgor (Farooq et al., 2009; Chen and Jiang, 2010); as a molecular chaperone stabilizing protein; as a ROS scavenger preventing oxidative burst (Szabados and Savoure, 2010); and stabilizing membranes thereby preventing electrolyte leakage (Yamada et al., 2005; Ashraf and Foolad, 2007). Consistent with the previous studies, the current study found proline was the most pronounced up-regulation metabolite in all the four genotypes responding to severer drought.

Despite its protective functions in response to environmental stresses, the toxicity of proline to plant growth has raised questions. Nanjo et al. (2003) isolated an Arabidopsis T-DNA-tagged pdh mutant that had a defect in proline dehydrogenase (AtProDH), which catalyzes the first step of proline catabolism. The pdh mutant showed hypersensitivity to exogenous application of 10 mM L-prolin, at which wild-type plants grew normally. Due to the toxicity effect, plants that were engineered to overaccumulate proline to enhance their tolerance to abiotic stress might not be resistant to field conditions (Deuschle et al., 2001; Rontein et al., 2002; Rizhsky et al., 2004). A large body of data on exogenous proline application showed enhanced stress tolerance of plants when proline is supplied at low concentrations, but toxic effects at higher concentrations (Hayat et al., 2012).

**Figure 6:**
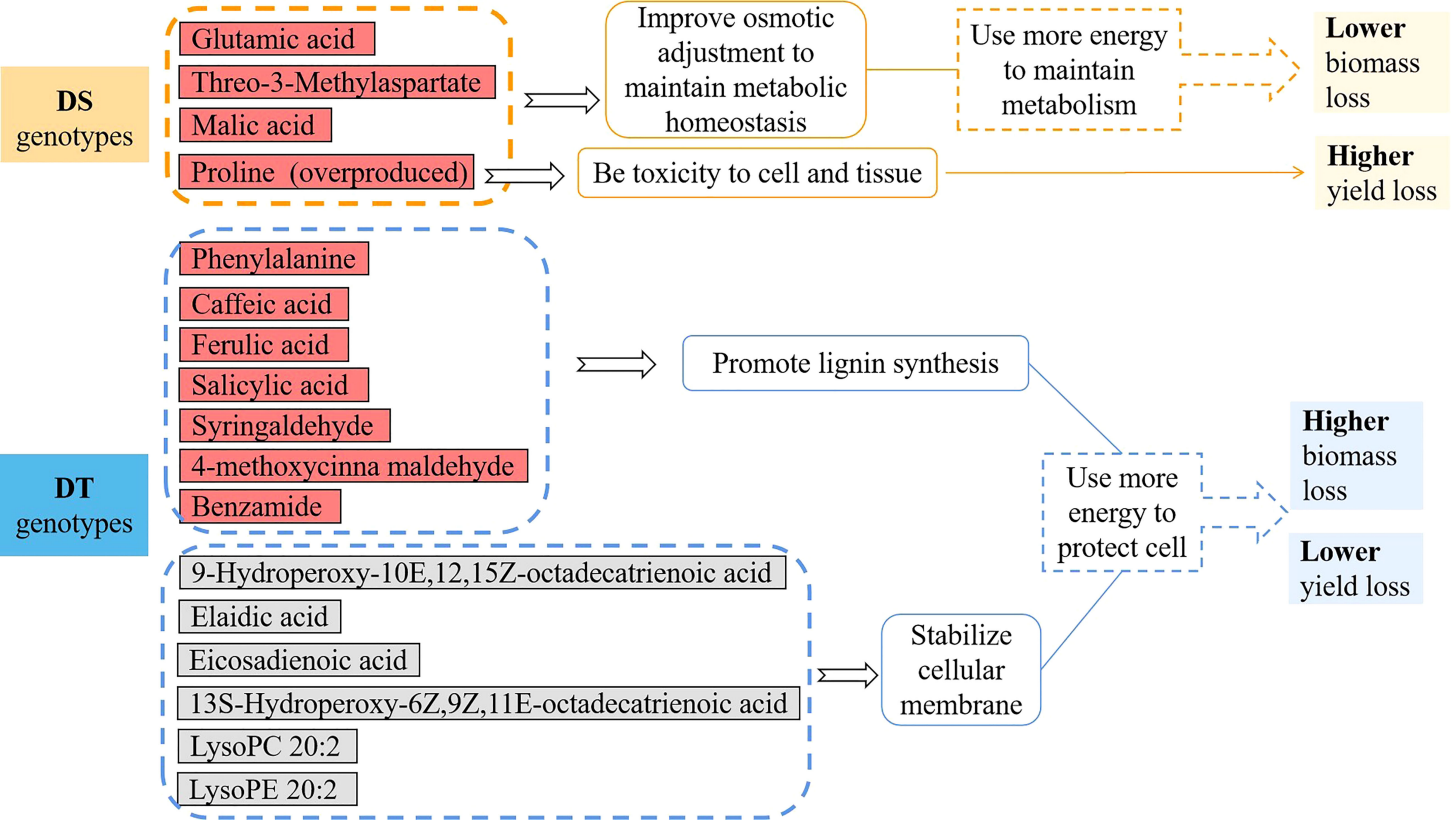
The differences of biomass loss, yield loss and metabolites between the drought tolerant (DT) dryland genotypes and drought sensitive (DS) irrigated genotypes under severer drought stress. In metabolic differences, the red color represents significant increase and gray color represents no significant change (p < 0.05) under severer drought stress.

As well known, proline levels are usually determined through the balance between biosynthesis and catabolism (Szabados and Savoure, 2010). Proline synthesis in the cytosol is stimulated by stress conditions whereas situations of stress recovery facilitate proline catabolism in mitochondria (Hossain et al., 2016). Proline can be toxic to cells, mainly if it is not appropriately removed from the cell system (Hellmann et al., 2000; Deuschle et al., 2001; Mani et al., 2002). In this study, difference in proline metabolic response among DS and DT genotypes was most pronounced. Seedlings of the two DS genotypes Lankaoaizao 8 and Zhengmai 9023 overaccumulated proline up to 25.4- and 14.13-fold. With such dramatic increase, proline metabolism in DS genotypes might be hard to restore the balance after released from stress, then result in proline toxicity to cell and tissue as a result, which ultimately led to the enormous yield loss (**Figure 6**).

### 4.3 Major metabolic differences between the seedlings of DT and DS genotypes in response to severer drought stress

#### 4.3.1 DS genotypes tended to up-regulated some amino acids and organic acids that help maintain cell metabolism

In the DS genotypes under severer drought stress, aside from proline overproduction, threo-3-Methylaspartate and glutamic acid were also remarkably up-regulated, which were not observed in the two DT genotypes (**Table 2**). Increased levels of some amino acids in stress sensitive genotypes were also observed in the previous studies. Some amino acids were increased in salt sensitive rice cultivars (Zuther et al., 2007) and *Lotus japonicus* (Sanchez et al., 2008), exposed to salt treatment; but did not in the halophyte *Limonium latifolium* (Gagneul et al., 2007), nor in the salt tolerant tree *Populus euphratica* (Brosche et al., 2005). Only proline and a few other amino acids (e.g. aspartate) were increased in these stress tolerant plants. (Widodo et al., 2009) also found increases in amino acids occurred earlier in salt sensitive than salt tolerant barley (*Hordeum vulgare* L.) cultivars. Thus increased levels of some amino acids might be an indicator of stress susceptibility for some plants.

On the other hand, these amino acids, acting as osmolyes, can improve cell hydration and maintain cell turgor at lower water potential so the plant can maintain metabolic processes in drying soil (Chen and Jiang, 2010). Moreover, L-threo-3-methylaspartate is mainly involved in C metabolism (Kyoto Encyclopedia of Genes and Genomes), and glutamate mainly acts to synthesize amino acids and organic acids (Fait et al., 2008; Sanchez et al., 2012), working as core regulators of C and N metabolism and involved in many metabolic networks (Less and Galili, 2008). Aside from the up-regulated amino acids, the amount of malic acid was increased in DS genotypes under severer drought stress, but did not change significantly in DT genotypes. Up-regulated malic acid was also reported in Bermuda grass under drought stress (Du et al., 2012). Malate plays vital roles in osmotic adjustment in guard cells, in pH balancing, and in stomatal functioning (Fernie and Martinoia, 2009; Xu et al., 2016). The up-regulated malic acid in DS genotypes might help maintain stomatal aperture for photosynthesis. Thus increases in these amino acids and organic acids in DS genotypes could contribute to maintaining cell metabolism and plant growth, which might explain their minor biomass loss after experiencing longer and severer drought, in comparison with DT genotypes (**Figure 6**).

#### 4.3.2 DT genotypes up-regulated beneficial amino acids and phenolic acids to enhance cell self-protection

In the present study, the only amino acid up-regulated in the two DT genotypes while remained unchanged in the two DS genotypes was phenylalanine. Phenylalanine increase in response to drought was also found in other plant species, such as wheat and lentil (Bowne et al., 2012; Muscolo et al., 2015). Increase in this amino acid might promote lignification and alleviate the damage of drought stress, as found in *Ctenanthe setosa* (Terzi et al., 2013). Additionally, phenylalanine increase can trigger the biosynthesis of phenolic acids through the cinnamic acid pathway (Nyarukowa et al., 2016). Consistently, the current study found in the DT genotypes under severer drought stress, the up-regulated phenylalanine was accompanied by increase in the following six phenolic acids caffeic acid, ferulic acid, salicylic acid, syringaldehyde, 4-methoxycinna maldehyde, and benzamide. Phenolics and phenolic acids increased under various environmental stress and function as important metabolites, as widely reported (Hura et al., 2008; Nakabayashi et al., 2014). In *Camellia sinensis*, caffeic acid level was significantly higher in the DT cultivars than in the DS cultivars (Nyarukowa et al., 2016). Ferulic acid was an important drought-responsive metabolite in wheat and barley (Hura et al., 2009; Piasecka et al., 2017). These phenolic acids could facilitate lignin synthesis to alleviate the damage of drought stress (Nyarukowa et al., 2016). It thus can be seen that the DT genotypes use more energy to synthesize beneficial amino acids and phenolic acids involved in some metabolic pathways, such as lignin synthesis, to protect the plant cells from drought damage (**Figure 6**).

Lipids are the most important component of cellular membranes and have a profound role for plant cells to resist environmental stresses. However, lipid peroxidation caused by overproduced ROS brought about lipid reduction, and in turn damage cellular membrane (Rinalducci et al., 2008; Farooq et al., 2009; Salehi-lisar et al., 2012). Huseynova et al. (2016) reported that the lipid peroxidation level was increased more significantly in susceptible wheat genotypes. In our research, there were six lipids down-regulated in DS genotypes under severer drought stress. While these lipids remained unchanged in DT genotypes, indicating the stabilized cellular membrane, which would be beneficial for the recovery and growth of plants after rehydration (**Figure 6**).

## 5 Conclusion

Comparison among dryland and irrigated genotypes, which contrast in water environment background, broke through the original cognition on the linkage between growth performances during drought stress with ultimate yield response. DS irrigated genotypes with high yield sensitivity to drought conversely showed minor biomass loss and higher Pn, also more dramatic proline increase during severer drought stress, in comparison with DT genotypes. Thus for evaluating drought tolerance of genotypes with different water environment background, evaluation system with yield being the core index remains to be the most robust up to date. Additionally, some superior DT dryland genotypes not only showed more stable yield after experiencing severer drought, but also showed excellent yield performance under WW conditions, confirming that a genotype can be in possession of both drought tolerance and high yield potential traits. It thus supports that superior dryland genotypes could be developed by improving superior irrigated genotypes in term of environmental adaptability and yield stability through breeding and selection. Profiling the metabolites in seedling leaves responding to severer drought might at least partly explain the genotypic differences in growth and yield responses to drought. The DS genotypes significantly increased some amino acids and organic acids to maintain cell metabolism and thus gained more biomass under severer drought stress. It might be ascribed to the overaccumulated proline that ultimately led to greater yield loss, which could not be appropriately removed after released from stress and become toxic to cell system. DT genotypes increased the beneficial amino acids and phenolic acids etc. to enhance cell self-protection to prevent plant cells from drought damage, thereby ultimately minimized yield loss.

## Data availability statement

The raw data supporting the conclusions of this article will be made available by the authors, without undue reservation.

## Author contributions

XZha performed the majority of experiments, collected the data, analyzed parts of the experimental data and results, and prepared the initial manuscript draft. YL, EL, XL, and FG performed the field experiment and prepare the related materials and methods part of the manuscript. RG performed the metabolomics data analysis and prepared the related part of the manuscript. ZY and SL provided the study materials, performed the filed experiment data analysis and participate in drafting of relevant content. XZho and XM conceived and designed the experiments and produced the final manuscript version. All authors contributed to the article and approved the submitted version.

## Funding

This work was supported by the grant from the National Key R & D Program (2021YFE0101300) and Central Public-interest Scientific Institution Basal Research Fund (BSRF202002).

## Conflict of interest

The authors declare that the research was conducted in the absence of any commercial or financial relationships that could be construed as a potential conflict of interest.

## Publisher’s note

All claims expressed in this article are solely those of the authors and do not necessarily represent those of their affiliated organizations, or those of the publisher, the editors and the reviewers. Any product that may be evaluated in this article, or claim that may be made by its manufacturer, is not guaranteed or endorsed by the publisher.

## Supplementary material

Supplementary Table 1 All metabolite profiles in the four wheat genotypes under well-watered condition and water stress condition.
