# Supplementary material for: Wheat genotypes with higher yield sensitivity to drought overproduced proline and lost minor biomass under severer water stress
Source: Front Plant Sci. 2022 Nov 29;13:1035038. doi: 10.3389/fpls.2022.1035038 (PMC9756133; doi:10.3389/fpls.2022.1035038)
Supplement: Supplementary file 2 [file Table_2.doc]

Table S2. Relative contents of metabolites in the four wheat genotypes and the significant difference between the two types of genotypes under soil water stress

| **Metabolites** | **Relative contents** | | | | **Significant difference** | | | |
| --- | --- | --- | --- | --- | --- | --- | --- | --- |
|  | **Jinmai 47** | **908216** | **Lankaoaizao 8** | **Zhengmai 9023** | **Jinmai 47-**  **Lankaoaizao 8** | **Jinmai 47-**  **Zhengmai 9023** | **908216-**  **Lankaoaizao 8** | **908216-**  **Zhengmai 9023** |
| **Amino acids** | | | | | | | | |
| Proline | 2.45E+06 | 3.17E+06 | 6.37E+06 | 4.11E+06 | ↑ | ↑ | ↑ | NS |
| Threo-3-Methylaspartate | 3.24E+05 | 4.18E+05 | 4.15E+05 | 3.85E+05 | NS | NS | NS | NS |
| Glutamic acid | 4.12E+06 | 4.43E+06 | 4.17E+06 | 5.54E+06 | NS | NS | NS | NS |
| Threonine | 1.95E+06 | 2.17E+06 | 1.59E+06 | 1.66E+06 | NS | NS | NS | NS |
| Valyl-L-Phenylalanine | 1.59E+06 | 1.11E+06 | 1.34E+06 | 1.32E+06 | NS | NS | NS | NS |
| Aspartic Acid | 6.40E+06 | 8.31E+06 | 4.31E+06 | 5.13E+06 | ↓ | NS | NS | NS |
| Phenylalanine | 8.90E+05 | 1.36E+06 | 1.07E+06 | 8.10E+05 | NS | NS | NS | ↓ |
| N-Acetyl-L-tyrosine | 3.60E+05 | 3.66E+05 | 3.05E+05 | 3.81E+05 | NS | NS | NS | NS |
| **Organic acids** | | | | | | | | |
| Trans-Citridic acid | 4.26E+06 | 5.45E+06 | 4.83E+06 | 4.59E+06 | NS | NS | NS | NS |
| 3-Hydroxyanthranilic acid | 5.31E+04 | 3.41E+04 | 3.69E+04 | 6.33E+04 | NS | NS | NS | ↑ |
| 2,2-Dimethylsuccinic acid | 7.49E+04 | 9.61E+04 | 3.03E+04 | 4.76E+04 | NS | NS | NS | NS |
| Malic acid | 4.10E+07 | 5.44E+07 | 6.11E+07 | 6.40E+07 | ↑ | ↑ | NS | NS |
| **Phenolic acids** | | | | | | | | |
| Caffeic acid | 2.01E+05 | 2.94E+05 | 1.57E+05 | 1.10E+05 | ↓ | ↓ | ↓ | ↓ |
| Ferulic acid | 1.93E+07 | 2.44E+07 | 7.60E+06 | 3.75E+06 | ↓ | ↓ | ↓ | ↓ |
| Salicylic acid | 5.55E+04 | 5.38E+04 | 4.25E+04 | 3.70E+04 | NS | ↓ | NS | ↓ |
| Syringaldehyde | 2.13E+05 | 2.16E+05 | 1.90E+05 | 1.86E+05 | NS | NS | NS | NS |
| 1. MethoxycinnaMaldehyde | 1.62E+05 | 1.59E+05 | 1.36E+05 | 1.39E+05 | ↓ | ↓ | ↓ | NS |
| Benzamide | 3.74E+05 | 4.07E+05 | 5.16E+05 | 4.54E+05 | NS | NS | NS | NS |
| 3,4-Dihydroxybenzeneacetic acid | 1.50E+05 | 1.09E+05 | 1.60E+05 | 1.54E+05 | NS | NS | NS | ↑ |
| Vanillin | 2.73E+06 | 2.78E+06 | 2.68E+06 | 2.52E+06 | NS | NS | NS | NS |
| Coniferyl alcohol | 4.67E+04 | 5.27E+04 | 5.46E+04 | 4.41E+04 | NS | NS | NS | NS |
| Methyleugenol | 2.81E+03 | 2.65E+03 | 6.13E+03 | 6.48E+03 | ↑ | NS | ↑ | NS |
| **Lipids** | | | | | | | | |
| 9-Hydroperoxy-10E,12,15Z-octadecatrienoic acid | 7.45E+05 | 7.23E+05 | 4.75E+05 | 4.44E+05 | ↓ | ↓ | ↓ | ↓ |
| Elaidic Acid | 5.19E+07 | 4.61E+07 | 3.43E+07 | 3.38E+07 | NS | NS | NS | ↓ |
| Eicosadienoic acid | 4.83E+06 | 3.85E+06 | 2.22E+06 | 2.51E+06 | NS | NS | ↓ | ↓ |
| 13S-Hydroperoxy-6Z,9Z,11E-octadecatrienoic acid | 1.18E+06 | 1.14E+06 | 7.31E+05 | 6.86E+05 | ↓ | ↓ | ↓ | ↓ |
| LysoPC 20:2 | 1.81E+04 | 1.03E+04 | 1.15E+04 | 9.72E+03 | NS | NS | NS | NS |
| LysoPE 20:2 | 3.86E+03 | 2.99E+03 | 2.15E+03 | 1.63E+03 | NS | NS | NS | ↓ |
| **Others** | | | | | | | | |
| 4-Methyl-5-  thiazoleethanol | 8.11E+04 | 1.02E+05 | 8.63E+04 | 7.58E+04 | NS | NS | NS | NS |
| Pinoresinol-4,4'-O-di-O-glucoside | 4.33E+05 | 3.68E+05 | 2.91E+05 | 4.05E+05 | NS | NS | NS | NS |
| Jaceosidin | 5.35E+06 | 4.60E+06 | 5.57E+06 | 6.34E+06 | NS | NS | NS | ↑ |
| Rhamnoside | 9.63E+05 | 9.30E+05 | 9.66E+05 | 9.53E+05 | NS | NS | NS | NS |
| Salcolin A | 1.17E+07 | 1.16E+07 | 1.15E+07 | 1.16E+07 | NS | NS | NS | NS |
| Choline | 1.27E+07 | 1.23E+07 | 1.13E+07 | 1.02E+07 | NS | NS | NS | NS |
| Adenosine 5'-monophosphate | 9.10E+04 | 8.31E+04 | 6.52E+04 | 4.82E+04 | NS | NS | NS | ↓ |
| Phenethylamine | 1.34E+06 | 1.41E+06 | 1.93E+06 | 1.70E+06 | ↑ | NS | ↑ | NS |

Note: NS means no significant difference, ↑ means up-regulated significantly and ↓ means down-regulated significantly at p < 0.05.
